# Does Environmental Policy Help Green Industry? Evidence from China’s Promotion of Municipal Solid Waste Sorting

**DOI:** 10.3390/ijerph18062799

**Published:** 2021-03-10

**Authors:** Di Chen, Yue Wang, Yang Wen, Honglin Du, Xue Tan, Lei Shi, Zhong Ma

**Affiliations:** 1 School of Environment and Natural Resources, Renmin University of China, Beijing 100872, China; chendi16@ruc.edu.cn (D.C.); wangyue_prc@ruc.edu.cn (Y.W.); 2020104564@ruc.edu.cn (H.D.); zhongma@vip.sina.com (Z.M.); 2 China Academy of Macroeconomic Research, Beijing 100038, China; wenyangniu@ruc.edu.cn; 3 Institute of Spatial Planning & Regional Economy, National Development and Reform Commission, Beijing 100038, China; 4 State Grid Energy Research Institute Co., LTD, Beijing 102209, China; tanxue@sgeri.sgcc.com.cn

**Keywords:** waste sorting, investor reaction, event study, DID, China

## Abstract

As municipal solid waste severely threatens human health and the ecological environment, since 2019, China has started to fully practice MSW sorting in all prefecture-level cities. In this paper, we apply the event study and difference-in-difference methods to investigate how China’s green policy of promoting MSW sorting influences listed waste sorting companies from the perspective of investors’ short-term and long-term reactions. This paper finds that investors are not sensitive to the introduction of MSW sorting in the short term, the new environmental policy does not relieve the financing constraints of related enterprises in the long run, and the financing constraints of private enterprises are stricter than those of state-owned enterprises. These findings indicate that China’s current encouragement of garbage sorting is not efficient enough as it has not brought benefits to the waste classification industry yet. More measures need to be taken to eliminate uncertainties in urban waste sorting. Our paper enriches the research on China’s waste sorting practices and provides new evidence of the effects of environmental policy on related firms from the perspective of green industry.

## 1. Introduction

With rapid industrialization and urbanization, municipal solid waste (hereinafter referred to as MSW) has become a significant environmental pollution issue and health threat to urban residents, especially in China and other developing countries [1,2,3]. From 2013 to 2019, urban household waste produced by large and medium-sized cities in China increased from 161.48 to 235.60 million tons at a rate of 6.55% per year, which approximated to the annual growth rate of China’s GDP [4]. The dilemma of “waste sieges”, which means cities are surrounded by accumulated waste in suburban or rural areas, has recently been a common problem among large and medium-sized cities in China [5,6,7]. 

Although China has made great sustained efforts to promote MSW sorting for many years [8,9], the effective implementation of MSW sorting has not achieved yet due to weak public awareness of solid waste recycling, insufficient coordination among government departments, and the inadequacy of related technology and infrastructure [10,11]. In recent years, as a response to the state environmental strategy for keeping the sky blue, water clear, and land pollution-free (which is known as the Strict Battle for Pollution Prevention and Control), China, thus reiterated the significance of MSW sorting and planned to practice MSW sorting in all prefecture-level cities [12]. To actively practice this environmental strategy, the Ministry of Housing and Urban-Rural Development of China (hereinafter referred to as the MHURD) introduced a plan in 2017 to require 46 key cities to first establish a basic system of laws and regulations on waste sorting by the end of 2020 [13]. In 2019, waste sorting has been promoted in all prefecture-level cities after being piloted in 46 cities, and the MHURD required all cities at the prefectural level or above to have their own household garbage sorting and disposal systems by 2025 [13,14]. Given this, MSW sorting has gradually been introduced into local legal systems, and many cities such as Shanghai, Beijing, Taiyuan, and Changchun have already enacted local decrees on garbage sorting since 2019 [15]. 

The promotion of urban waste sorting has ignited heated discussions in China and has been an important issue of concern to scholars for quite a long time [5,16]. The current literature has investigated China’s waste sorting practices from many perspectives. Aside from focusing on assessing the environmental impact of MSW sorting [17,18,19] and evaluating the performance of waste sorting facilities or systems in specific areas [20,21,22], researchers have also conducted studies on how to improve the efficiency of the management of MSW sorting. In this field, scholars have analyzed the engagement factors for the promotion of MSW sorting [23,24] and identified that law enforcement, financial support, and public awareness could help to achieve the effective reduction and recycling of municipal waste [25,26,27,28]. However, few studies focus on how the promotion of garbage classification in urban areas influences the related industry, especially the firms whose main business is trash sorting. To ensure the waste sorting at the city level could gradually be achieved, the MHURD identified that local governments should enhance the construction of waste sorting facilities and expand the supply of services regarding waste management [29]. Considering that the waste sorting industry is the main provider of goods and services for municipal garbage separation, these supporting measures above are believed would increase the market demand of the waste management industry and further influences its development [30]. With years of efforts to explore effective MSW management in pilot cities, China’s plan for practicing MSW sorting is widely regarded as the start of strengthening public engagement in MSW sorting, which would lead China into a new era of stringent urban MSW management [31]. Therefore, by measuring the impacts on the financial performance and the capability of the waste sorting industry, which are the key indicators to reflect the investor confidence in specific industries and the extent of public awareness of MSW sorting, the effectiveness of waste sorting promotion could be estimated from a new horizon [32]. In addition, though studies on the effect of environmental policy from the perspective of polluting firms have aroused discussions for years [33,34], few researchers have focused on the impact that environmental regulation has on green enterprises. To bridge this research gap, this study estimates how China’s promotion of MSW sorting influences MSW sorting enterprises from two perspectives of the stock market’s short-term reaction and investors’ long-term preference. This study contributes to the literature in two main ways: First, we enrich studies on whether China’s MSW sorting policy encourages the public to realize the importance of garbage classification by analyzing the changes in investors preference for waste sorting firms; Second, we take the waste sorting industry as our main sample, examining the impact of urban waste sorting policy on related firms, and providing new evidence on the impact of environmental protection policies on green industry in the context of developing countries.

The remainder of the paper is organized as follows. Section 2 provides a literature review of related research. Section 3 introduces the data and methodology. Section 4 reports the results. Section 5 presents the discussion, and finally, we conclude our findings in Section 6.

## 2. Literature Review

Policies are proven to have significant impacts on corporate financing and investment activities [35]. As some of the literature identified, environmental regulations could affect an investor’s willingness to invest [36,37]; the implementation of waste sorting could also influence green industry enterprises, especially those companies whose primary business is MSW sorting [38]. In particular, by applying the method of event study, which is a key research method in analyzing the immediate impact of specific events on related firms, scholars have conducted abundant studies on the examination of how the stock market reacts to environmental regulation. However, most of the current studies focus on the stock market’s disciplinary effect or environmental policy’s deterrent effect on heavily polluting enterprises instead of analyzing the benefits that green industry enterprises may receive from environmental regulation [39,40]. Many scholars believe it is wise for enterprises to attach importance to their environmental performance since they estimate that it could help enterprises to receive a positive market reaction or reduce negative effects when environmental regulations are introduced [41,42]. Judging from that, a broad consensus that the introduction of new environmental regulation could potentially increase green industry enterprises’ return by boosting the market demand for pollution abatement goods and services and environmental protection technologies are reached among researchers. Even though there are some studies that indirectly investigated whether environmental policies could influence green firms by analyzing changes in the environmental performance [43,44] or the productivity [45,46] of polluting companies, the effect of environmental policy on the green industry has still been less investigated. Therefore, this paper intends to adopt an event study but with a focus on the short-term policy effect on the green industry (waste sorting industry) in order to add new empirical evidence on this issue.

Additionally, investors’ willingness to invest could vary with the change of times according to current findings on polluting firms. Consistent with the traditional view that environmental protection usually comes at an additional cost imposed on firms, and affects their competitiveness, some studies reveal that environmental regulations have an adverse effect where investors will reduce investment in polluting firms when higher pollution abatement costs would significantly lower productivity levels [47]. In contrast, a growing group of scholars observed the positive effects that environmental policies have on firms in the long term and reported that environmental regulations can improve the competitiveness and productivity of enterprises by encouraging innovation in pollution control (known as the Porter Hypothesis) [48,49]. As improvement in the polluting firm’s productivity is normally related to expanding investment in pollution abatement or clean production, the influence of environmental regulations on the green industry may also be different in the short and long term. Scholars note that investors are generally considered to have limited attention and have an incentive to focus on general summary statistics rather than individual pieces of information [50]; therefore, for the MSW sorting industry, some studies estimate a supportive policy related to this industry’s development could theoretically offer a positive signal to investors, such as encouraging more investors to invest in related enterprises, thus gradually helping to ease the financing constraints of the industry [51,52,53]. However, some researchers [24] doubt that current public perception and behaviors towards waste sorting in China are satisfactory enough to generate sufficient confidence in the good implementation of MSW sorting and to help relieve the industry’s financing problem in the short run. Hence, aside from using an event study to measure the short-term effect on waste sorting firms, this paper plans to further estimate the changes in investment preferences of investors in the long term so as to better investigate whether the promotion of MSW sorting in all prefecture-level cities benefits the waste sorting industry.

## 3. Materials and Methods 

### 3.1. Methodology

#### 3.1.1. Event Study

Based on the Effective Market Hypothesis, the event study method is a general approach to assess the effects of specific events on stock markets by examining the stock’s return(value) before and after an unexpected event or news [54]. The specific procedures of event study methodology used in this study are as follows:

(1) Definition of the event 

To ensure our results are accurate, we select 26th April 2019, the date that the MHURD declared to start MSW sorting in all prefecture-level cities, as the event date (T_0_) in our study [55]. 

(2) Definition of the event window and estimation window 

The event window is the period within which investors’ response to the inspection can be measured to estimate the short-term reaction to the stock market; we define [T_0_-10, T_0_+10] as the event window according to previous literature [56]. The estimation window is defined as the period within which a stock’s normal relation with the market can be estimated. Previous studies chose estimation windows ranging from 90 to 200 days [57]. To ensure our estimation window is efficient enough to calculate meaningful estimates of normal returns and avoid influences from other events [58], we set 150 trading days before the event window ([T_0_-160, T_0_-10]) as our estimation window. 

(3) Estimation of normal returns

Normal returns represent the returns that would have been realized if the analyzed event would not have taken place [59]. In this paper, we apply the market model to estimate normal returns (shown in Equation (1)) as this model has been commonly used in previous research [60,61]:

(1) $$R_{it}=\alpha_{i}+\beta_{i}R_{mt}+\varepsilon_{it},\;t\in \left[-160,10\right]$$

where $R_{it}$ and $R_{mt}$ denote the real return of stock i on day t and the market return on day t, respectively. To obtain the parameters $\alpha_{i},\beta_{i}$ of the market model, the Shanghai Shenzhen CSI 300 Index is employed as the proxy for the market index $R_{mt}$, and $\varepsilon_{it}$ represents the regression residual.

(4) Calculation of abnormal returns

The abnormal return (AR, hereafter) and the cumulative abnormal return (CAR, hereafter) are used to measure stock market reactions to specific events. Among them, the AR is calculated by deducting the normal returns from the actual returns of the stocks and is regarded as the stock market’s reaction to the arrival of an event [62]. The CAR is the total of all abnormal returns and could reflect the total impact of an event over a particular period of time. Specific models of calculating those indicators are the following:

(2) $${\mathrm{AR}}_{it}=R_{it}-\left(\hat{\alpha_{i}}+\hat{\beta_{i}}R_{mt}+\varepsilon_{it}\right),\;t\in \left[-10,10\right]$$

(3) $${\mathrm{CAR}}_{it}=\overset{N}{\underset{i=1}{\sum }}{\mathrm{AR}}_{it},t\in \left[-10,10\right]$$

where ${\mathrm{AR}}_{it}$ demonstrates the abnormal return of enterprises i on day t, and the sub-model $\hat{\alpha_{i}}+\hat{\beta_{i}}R_{mt}+\varepsilon_{it}$ represents the daily expected normal return. ${\mathrm{CAR}}_{it}$ is the cumulative abnormal return of firm i on day t, indicating the cumulative influence of an event over a time period. Additionally, to estimate the reactions of the stock market in general to the event, we use Equation (4) to calculate the cumulative average abnormal return (CAAR):

(4) $$CAAR_{\left[t_{1},t_{2}\right]}=\overset{t=t_{2}}{\underset{t=t_{1}}{\sum }}AAR_{t}=\overset{t=t_{2}}{\underset{t=t_{1}}{\sum }}\frac{1}{N}\overset{N}{\underset{i=1}{\sum }}AR_{it}$$

where $AAR_{t}$ is the average abnormal return of the stock market on day t and $CAAR_{\left[t_{1},t_{2}\right]}$ denotes the stock market’s cumulative average abnormal return during [t_1_, t_2_].

(5) Significance test

#### 3.1.2. Difference-In-Differences (DID)-Based Propensity Score Matching (PSM)

This study employs the PSM-DID method to quantitatively examine whether and to what extent China’s promotion of MSW sorting influences the financing constraints of the waste sorting industry. The difference-in-differences (DID, hereafter) method is widely acknowledged as the best method to evaluate the causal effects of specific external shocks, such as policy implementation [63,64,65,66]. Compared to ordinary regression (e.g., ordinary least square method), the DID method could divide the whole sample into the treatment group and the control group, and clearly identify the difference between the treatment group and the control group before and after the policy [67]. Therefore, the basic regression model in our study is constructed as follows [68,69,70]:

(5) $$FC_{i,t}=\alpha +\beta_{0}Treated_{i}\times Period_{t}+\beta_{1}Treated_{i}+\beta_{2}Period_{t}+\theta \sum X_{i,t}+\varepsilon_{i,t}$$

where $FC_{i,t}$ refers to the financing constraints of firm i at time t. We use the Size-Age index (hereinafter referred to as SA Index) to measure $FC_{i,t}$ based on the current previous literature [71,72], and the calculation formula of the SA index is (−0.737 × Size) + (0.043 × Size^2^) − (0.040 × Age), where Size is the log of total assets and Age is the number of years the enterprise has been listed. The higher the SA index, the more serious the financing constraints are. $Treated_{i}$ indicates firm i‘s industry, i.e., $Treated_{i}$ = 1 if firm i is an MSW sorting firm and =0 if firm i is a non-MSW sorting firm. $Period_{t}$ indicates the post-treatment period, i.e., $Period_{t}$ = 1 if t ≥ 2019-07-01 and =0 otherwise. $\varepsilon_{i,t}$ is the error term. $X_{i,t}$ denotes control variables, according to the available research [73,74]; the control variables in our study are as follows: size (represented by the natural logarithm of total assets), ownership ($Ownership_{i}$ = 1 if firm i is a state-owned enterprise and =0 otherwise), the proportion of tangible assets to total assets (asset tangibility), the growth rate of business income (growth), Current ratio, Asset-liability ratio (Lev), the ratio of institutional ownership (instinv), the return on assets (hereinafter referred to as ROA, usually calculated by dividing a company’s net income by total assets), the return on stockholders’ equity(hereinafter referred to as ROE, calculated by dividing net income by shareholders’ equity), and the proportion of net cash flow to total assets (cashflow).

However, a concern with the DID approach is that the estimation results can be biased if the treatment group and control group are not stochastically selected. Accordingly, scholars suggest using the propensity score matching (PSM, hereafter) method, which has been widely used in studies on policy effects since its introduction, to handle the endogenous problems caused by selection bias [75,76,77]. The main steps of this method involve: (1) Estimating a logit or other discrete choice model of program participation; (2) Defining the region of common support and balancing tests; (3) Matching pairs; and (4) Calculating the average treatment impact. In this study, the matching model is given by:

(6) $$Logit\left(treated_{it}=1\right)=\varphi \left(X_{i,t}\right)$$

where $X_{i,t}$ denotes matching variables and are chosen from our control variables in Equation (6). 

### 3.2. Sample and Data Source

We obtained firm-level daily stock return and semi-annual financial data for the event study and DID study, respectively. All data are collected from the China Stock Market and Accounting Research (CSMAR) database.

Our sample includes all listed enterprises in the Shanghai Exchange Market and the Shenzhen Exchange Market and is divided into different groups. In our event study part, we mainly compared the abnormal returns of MSW sorting enterprises, other green industry enterprises where the primary business is not MSW sorting, and heavily polluting enterprises. For the DID study, we divided our sample into two groups, namely the treatment group (MSW sorting enterprises), and the control group (listed enterprises whose businesses are unrelated to waste sorting). The MSW sorting enterprises and other green industry enterprises were chosen according to the list of enterprises offered by the Tonghuashun (iFinD) database, which is one of the most reliable financial databases among Chinese investors [78]. By Tonghuashun’s definition, enterprises whose primary business is waste management including waste sorting, transfer, and incineration, were selected as MSW sorting enterprises; enterprises whose main business related to environmental protection and energy-saving but not waste management were defined as the other green industry enterprises.

Additionally, to maintain the accuracy of our estimation, we excluded special treatment enterprises (e.g., ST, PT, *ST, etc.) as they had continuous worse accounting performance and stocks without enough normal trading days (less than 150 trading days) during the event window. The final sample of our study contained 2244 stocks, and we used winsorization to mitigate the effect of extreme values.

## 4. Results

### 4.1. Investor Reaction to Mandatory MSW Sorting

**Figure 1:**
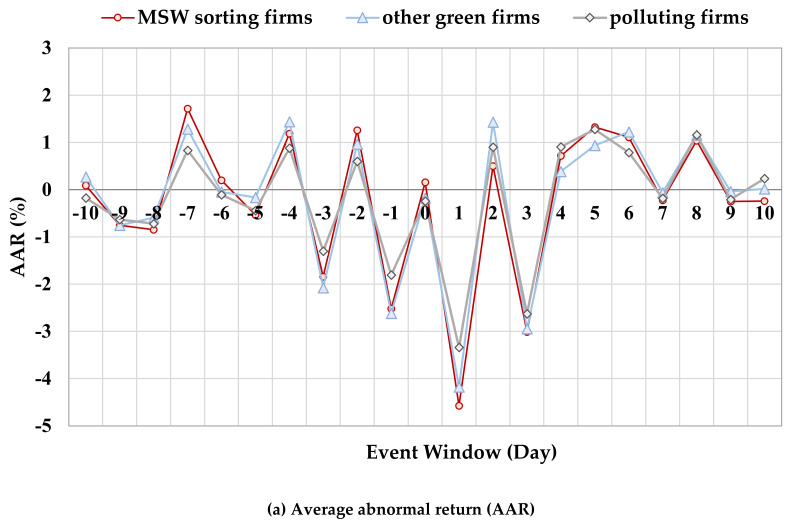

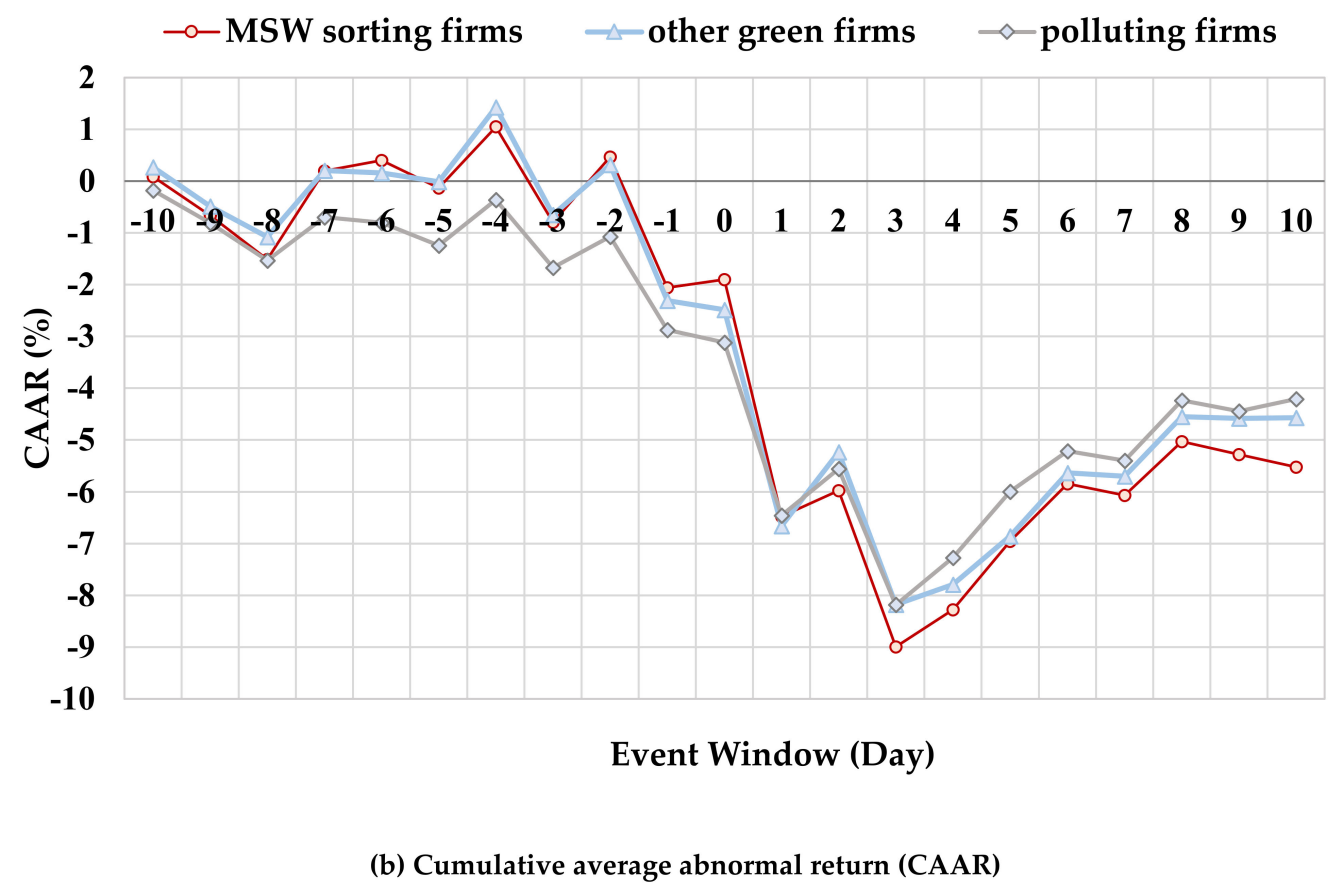
Trends of abnormal returns.

Figure 1 reports the market reaction to China’s promotion of MSW sorting in all prefecture-level cities during the event window [T_0_ − 10, T_0_ + 10]. The X-axis indicates the number of trading days during the event window, whereas the Y-axis represents the average abnormal return (AAR, hereafter) and average cumulative abnormal return (CAAR, hereafter) of enterprises. During the event window, the three groups of enterprises show the same fluctuation pattern, indicating that the promotion of MSW sorting does not increase investors’ preference for the MSW sorting industry and does not benefit this industry in the short term.

Table 1 presents the descriptive statistics of AARs and CAARs among MSW sorting enterprises, other green industry enterprises, and heavily polluting industry enterprises. For all kinds of enterprises, CAARs are statistically significant after the event date, showing the whole stock market responds negatively to the promotion of MSW sorting. The AARs of heavily polluting enterprises in the event window [T_0_ − 10, T_0_ + 10] are essentially all statistically significant, while the AARs of green industry enterprises including MSW sorting enterprises are mainly statistically significant after the event date. Investors are more sensitive to the potential returns loss of heavily polluting industry than the possible benefits to the MSW sorting industry upon the announcement of MSW sorting policies, which is consistent with many research findings where investors tend to respond negatively to environmental events and this negative response could be more severe and obvious towards heavily polluting enterprises [79,80,81].

### 4.2. Mandatory MSW Sorting’s Effects on Firm Financing Constraints

We observed that stock market does not have a positive reaction to China’s promotion of MSW sorting from our event study. We attempted to estimate the longer term effect that this promotion has on MSW sorting enterprises by using the difference-in-differences method. To minimize the selection bias caused by the different initial conditions between the treatment group and the control group, we first apply the propensity score matching (PSM) method to “balance” these two groups according to a set of baseline characteristics. Table 2 performs the PSM balance test. As shown in this table, all absolute values of normalized bias value are less than 10 after matching. Meanwhile, the t-statistics after matching were not significant, implying there is no systematic difference between the experimental group and the control group, and that our matching estimation results are suitable and reliable [76].

Based on our PSM results, the effect that China’s promotion of MSW sorting has on financing constraints at the firm level is shown in Table 3. As observed from our results, the SA Index of MSW sorting enterprises did not significantly decrease with the promotion of China’s MSW sorting (see model DID and PSM-DID in Table 3), revealing this promotion has no influence on the investment preferences of investors and the financing constraints of concerning firms. A lack of confidence from investors could be one of the reasonable explanations for the absence of a positive market reaction. According to Wu et al’s text mining study on Shanghai’s mandatory MSW sorting program [16], the proportion of positive emotions from the public towards the practice of MSW sorting is only slightly higher than that of negative emotions during the early implementation of the MSW sorting policy (July) or 4 months after its implementation (November). Their further analysis of negative topics showed the public’s awareness of MSW sorting was still too weak to enhance investors’ confidence in the implementation of MSW sorting.

Better asset tangibility, enterprise growth, higher asset-liability ratio, ROA, and institutional ownership ratio could help in relieving financing constraints. The financing constraints of state-owned enterprises (SOEs, hereafter) are generally lower than those of non-state-owned enterprises (non-SOEs, hereafter), supporting the current findings that investors have a higher preference to SOEs because of their political connections with governments [82,83]. 

In the robustness test, we re-estimate the effect that the enactment of China’s promotion of MSW sorting has on enterprises’ financing constraints by changing the treatment group (see Robustness Test (1) in Table 3) and the enactment date of China’s promotion of MSW sorting (see Robustness Test (2) in Table 3). Our results suggest that there is no evidence that China’s promotion of MSW sorting could help to relieve MSW sorting enterprises’ financing constraints. The relationships between control variables and financing constraints remain the same, confirming our previous finding that SOEs are more competitive in financing compared to non-SOEs. 

## 5. Discussion

With the development of urbanization and the improvement of living standards, MSW disposal has become one of the most critical public health issues challenging developing countries and regions. Since effective domestic waste sorting contributes to pollution abatement and public health, studies in the literature on how to achieve operative waste management have increased in recent years. 

Using various methods including the real options approach [84,85], system dynamics model [7,86], and exploratory factor analysis with fuzzy set theory [87], existing research has carried out beneficial assumptions analysis and case studies on how to effectively promote MSW management. Compared to current findings, this paper emphasizes the empirical examination of the specific effect that waste sorting policy has on urban garbage sorting firms. By measuring the alteration of investor attitude towards the MSW sorting industry, we found that unlike the typical disciplinary response to polluting enterprises when an environmental regulation is announced or even an environmental incident, investors did not give a positive reaction to the green industry when facing a new environmental policy, indicating the stock market is not sensitive enough to the green industry when an environmental policy is introduced.

Our findings provided empirical evidence that China’s current urban waste sorting practices are not efficient enough, as the main-related industry has not been influenced yet, implying that more measures need to be taken so that the threatens from waste disposal pollution could truly be avoided as much as possible. At present, uncertainty in MSW sorting is the main hindrance for the practice of trash sorting in urban areas [88] and could be the reason why investors do not have a positive preference for the MSW sorting industry, even though China declared the implementation of MSW sorting in all prefecture-level cities. Unlike other environmental regulations which have a clear mechanism of rewards and penalties or a strict supervision system for polluting enterprises [89,90] and despite the MHURD claiming that China required all municipal cities to practice MSW sorting, there is no formal strict national law to regulate urban waste sorting, as well as providing a sufficient incentive for classifying waste or a deterrent effect for not sorting garbage. Therefore, the present promotion of MSW sorting is inadequate for convincing residents, including investors, that the MSW sorting policy would be fully implemented or potentially help the MSW sorting industry’s development [16]. To tackle this, governments should strengthen their determination on mandatory MSW sorting. By introducing this regulation and improving education on MSW sorting among citizens, the effectiveness of MSW sorting policy could be ensured and enhanced [15,28], thus truly boosting the private sector’s confidence in investing in the MSW sorting industry.

Our study on the changes of financing constraints at the firm level identifies that the financing constraints of non-SOEs are stricter than SOEs, indicating that more financial support should be given to private enterprises. As the main supplier for the goods and services of environmental protection, the green industry plays a vital role in national and regional pollution abatement [91,92]. In China, though non-SOEs occupy a major proportion of the market and own 50% more green patents and technologies than SOEs [93], non-SOEs still face severe financing constraints due to their weak connection with governments and limited access to environmental protection projects [94]. Fortunately, China has recently started to take measures in easing financing difficulties among private enterprises. The mixed-ownership reform in the green industry, which is aiming to establish a more efficient and modern enterprise system [95], is believed to benefit non-SOEs’ financing and development. By bringing in and mixing multiple types of investors and exploring flexible and market-based salary systems, mixed-ownership reform can help to overcome the shortcomings that SOEs have such as the weaker motivation to innovate and lower efficiency [96], whereas non-SOEs could gain the synergy of both state support and private business strength, therefore enhancing their sustainable development. The green industry in China has already provided evidence that non-SOEs who accepted support from SOEs had successfully solved their financing difficulties and were recovered obviously, such as Beijing SPC Environment Protection Tech Co Ltd (002573)’s net cashflow in 2019 increasing 26.7%, where this enterprise just achieved its mixed-ownership reform in 2019.

## 6. Conclusions

MSW sorting has been and will continue to be a major issue in the urbanization process of China. The implementation of waste sorting in urban areas could not only be the key to efficient resource recycling but also offer new support to the MSW sorting and related industries development by offering potential business opportunities. By taking China’s promotion of MSW sorting as our study case, we firstly used the event study to estimate the investors’ short-term reaction and observed that investors do not respond positively to the MSW sorting industry’s returns. We further used the DID method to explore the long-term influence that China’s promotion of MSW sorting had on investor preferences and found that this promotion did not mitigate the financing constraints of waste sorting enterprises, and the financing constraints for private firms are more severe than SOEs.

We come to the conclusion that China’s current waste sorting practices in urban areas are not efficient enough, as investors have not been encouraged to invest in green stocks when this policy came out. To enhance residents’ engagement in MSW sorting and trigger the stock market’s investment preference in MSW sorting, China needs to take measures to ease the uncertainties in the current management of waste sorting including enacting related national laws, clarifying the mechanism of rewards and punishments.

Furthermore, more support needs to be given to non-SOEs in the MSW sorting industry. Except for preferential policies such as opening more access to energy-saving and environmental protection projects, we suggest China continue its mixed-ownership reform in the MSW sorting industry, thus easing the MSW sorting industry’s financing constraints, promoting the sustainable process of MSW management.

This study also has limitations. First, though the event study method has been commonly applied to measure the impact of events on investors, the accuracy of the study results relies on the efficiency of the stock market, whereas many event studies on the China stock market identified that the current capital market in this country is still underdeveloped and stock prices may be limited to reflect firms’ true value. Therefore, further interpretation and examination should be cautiously conducted. Second, this study assumes there is no distinction between retail investors versus institutional investors, while in reality, institutional investors could have different sentiments about the prospect of policy-related firms since they usually own more resources and could be better informed than retail investors. The challenge that needs to be further addressed is to separate the impacts of institutional investors from retail investors. In addition, this paper does not estimate the long-term market reaction to the promotion of MSW sorting from the perspective of the buy-and-hold abnormal return (BHAR) approach due to insufficient data availability. Related data and the long-term changes in investors’ preference for waste sorting firms could be further collected and confirmed in future studies. 

## Figures and Tables

**Table 1:** Display of abnormal returns.

Day	Average Abnormal Return (AAR)			Cumulative Average Abnormal Return (CAAR)		
	MSW Sorting Firms	Other Green Firms	Polluting Firms	MSW Sorting Firms	Other green Firms	Polluting Firms
−10	0.0885	0.2693	−0.1748 **	0.0885	0.2693	−0.1748 **
−9	−0.7557 ***	−0.7532 ***	−0.6322 ***	−0.6672 **	−0.4839	−0.807 ***
−8	−0.8481 ***	−0.5939 **	−0.723 ***	−1.5153 ***	−1.0779 ***	−1.53 ***
−7	1.7151 ***	1.281 ***	0.8352 ***	0.1999	0.2031	−0.6948 ***
−6	0.1997	−0.0482	−0.1071	0.3996	0.1549	−0.8019 ***
−5	−0.534 **	−0.1671	−0.4435 ***	−0.1344	−0.0121	−1.2455 ***
−4	1.1841 ***	1.4397 ***	0.8806 ***	1.0497	1.4276 *	−0.3648
−3	−1.8446 ***	−2.0771 ***	−1.303 ***	−0.7949	−0.6496	−1.6678 ***
−2	1.2585 ***	0.9635 ***	0.5991 ***	0.4636	0.3139	−1.0687 ***
−1	−2.5217 ***	−2.6202 ***	−1.8055 ***	−2.0581 **	−2.3063 ***	−2.8742 ***
0	0.1552	−0.1798	−0.2403 ***	−1.9029 *	−2.4861 ***	−3.1145 ***
1	−4.5748 ***	−4.177 ***	−3.3403 ***	−6.4777 ***	−6.6631 ***	−6.4548 ***
2	0.4984	1.4288 ***	0.8997 ***	−5.9793 ***	−5.2343 ***	−5.5551 ***
3	−3.0137 ***	−2.9428 ***	−2.6257 ***	−8.993 ***	−8.1771 ***	−8.1808 ***
4	0.7138 **	0.3833	0.9061 ***	−8.2793 ***	−7.7938 ***	−7.2747 ***
5	1.3256 ***	0.9335 ***	1.2766 ***	−6.9537 ***	−6.8603 ***	−5.9981 ***
6	1.1089 ***	1.2247 ***	0.785 ***	−5.8447 ***	−5.6357 ***	−5.2131 ***
7	−0.225	−0.0664	−0.1826 ***	−6.0697 ***	−5.702 ***	−5.3957 ***
8	1.0379 ***	1.1546 ***	1.1615 ***	−5.0318 ***	−4.5474 ***	−4.2342 ***
9	−0.2494	−0.0377	−0.2078 ***	−5.2812 ***	−4.5851 ***	−4.442 ***
10	-0.2412	0.0152	0.2364 ***	−5.5224 ***	−4.5699 ***	−4.2056 ***

*** *p* < 0.01, ** *p* < 0.05, * *p* < 0.1.

**Table 2:** Propensity score matching (PSM) balance test.

Variable	Unmatched	Mean		%Bias	%Reduct | Bias |	*t*-Test	
	Matched	Treated	Control			*t*	*p* > | *t* |
size	U	22.623	22.485	11.4	55.0	0.85	0.394
	M	22.623	22.684	−5.1		−2.29	0.771
ownership	U	0.418	0.386	6.5	−87.6	0.53	0.593
	M	0.418	0.358	12.1		0.71	0.482
asset tangibility	U	32.638	43.092	−47.2	93.6	−3.85	0.000
	M	32.638	31.964	3.0		0.17	0.862
growth	U	19.095	53.706	−1.5	83.1	−0.09	0.931
	M	19.095	24.941	−0.3		−0.34	0.737
current ratio	U	1.664	2.414	−8.1	99.3	−0.47	0.636
	M	1.664	1.670	−0.1		−0.02	0.985
lev	U	50.463	43.234	37.6	72.7	2.92	0.003
	M	50.463	52.435	−10.3		−0.59	0.559
instinv	U	36.432	42.426	−26.1	88.1	−2.15	0.032
	M	36.432	35.717	3.1		0.19	0.849
ROA	U	3.001	3.995	−15.7	57.9	−1.32	0.188
	M	3.001	3.420	−6.6		−0.33	0.742
ROE	U	2.222	4.570	−15.2	84.4	−1.18	0.237
	M	2.222	1.856	2.4		0.09	0.926
cashflow	U	0.007	0.008	−1.4	−673.7	−0.10	0.923
	M	0.007	−0.001	10.7		0.74	0.459

**Table 3:** Results and robustness test.

Variable	Model (1) DID	Model (2) PSM-DID	Model (3) Robustness Test	Model (4) Robustness Test
treated*period	0.014 (0.23)	0.015 (0.25)	−0.003 (−0.13)	0.013 (0.29)
period	−0.183 *** (−16.32)	−0.183 *** (−16.19)	−0.182 *** (−12.72)	−0.189 *** (−22.51)
treated	0.037 * (1.94)	0.039 ** (2.04)	0.031 *** (4.40)	0.036 * (1.79)
size	1.216 *** (277.25)	1.217 *** (274.07)	1.216 *** (278.46)	1.222 *** (278.44)
ownership	−0.407 *** (−52.65)	−0.408 *** (−52.14)	−0.408 *** (−52.67)	−0.410 *** (−53.35)
asset tangibility	−0.001 *** (−3.02)	−0.001 *** (−2.89)	−0.001 ** (−3.06)	−0.001 *** (−3.14)
growth	0.000 (0.50)	0.000 (−1.52)	0.000 (0.41)	0.000 (0.41)
current ratio	0.000 (−0.45)	0.008 *** (3.46)	0.000 (−0.59)	0.000 (−0.61)
lev	−0.005 *** (−16.33)	−0.005 *** (−14.48)	−0.005 *** (−16.37)	−0.005 *** (-16.86)
instinv	−0.002 *** (−9.56)	−0.001 *** (−8.74)	−0.002 *** (−9.71)	−0.002 *** (−9.62)
ROA	−0.004 *** (−5.41)	−0.004 *** (−4.47)	−0.004 *** (−5.32)	−0.005 *** (−6.48)
ROE	0.000 (−0.20)	0.001 (1.22)	0.000 (−0.23)	0.000 (0.49)
cashflow	−0.067 (−1.64)	−0.065 (−1.55)	−0.067 (−1.62)	−0.081 *** (−1.99)
Constant	−22.606 *** (−234.35)	−22.647 *** (−230.7)	−22.607 *** (−234.62)	−22.654 *** (−235.6)
Number of Obs	22440	22130	22440	22440
R-squared	0.902	0.901	0.902	0.903
Prob > F	0.000	0.000	0.000	0.000

*** *p* < 0.01, ** *p* < 0.05, * *p* < 0.1.

## Data Availability

Restrictions apply to the availability of these data. Data was obtained from the China Stock Market and Accounting Research Database and are available at https://www.gtadata.com/ with the permission of the China Stock Market and Accounting Research Database.
